# The Regularity of the Site of Impaction in Recurrent Gallstone Ileus: A Systematic Review and Meta-Analysis of Reported Cases

**DOI:** 10.1155/2021/5539789

**Published:** 2021-12-02

**Authors:** Nasser A. N. Alzerwi, Bandar Idrees, Saeed Alsareii, Yaser Aldebasi, Afnan Alsultan

**Affiliations:** ^1^Department of Surgery, College of Medicine, Majmaah University, Ministry of Education, Al-Majmaah City, 11952, P.O. Box 66, Riyadh, Saudi Arabia; ^2^Gastrointestinal, Biliopancreatic, and Minimally Invasive Surgery at Department of Surgery, Prince Sultan Military Medical City in Riyadh, Makkah Al Mukarramah Rd, As Sulimaniyah, Riyadh 12233, Saudi Arabia; ^3^Department of Surgery, College of Medicine, Najran University, Najran, Saudi Arabia; ^4^Board Certified General Surgeon, Department of Surgery, King Salman Hospital in Riyadh, Riyadh, Saudi Arabia; ^5^Resident in Training, Department of Surgery, King Saud Medical City, Riyadh, Saudi Arabia

## Abstract

**Objective:**

Due to the rarity of recurrent gallstone ileus (RGSI), its epidemiological and clinical features are elusive. With a focus on mortality and the site of impaction, this study consolidates the key clinical characteristics of index GSI (IGSI) and RGSI.

**Methods:**

A meta-analysis of cases reported on RGSI was performed. Risk factors for mortality and site of impaction were examined, and a subgroup analysis was performed for age, sex, and site of impaction (jejunum, ileum, or others).

**Results:**

In the final analysis, 50 (56 individual cases) studies were included. The paired data for the site of impaction was available for 45 patients. Women accounted for 87.3% of all RGSI cases included in the pooled analysis. The median age (interquartile range, IQR) of the patients was 70 (63–76) years, and the median time of recurrence (IQR) was 20.5 (8.5–95.5) days. The overall mortality rate was 11.8%, without correlation between the mortality rate and age, the time of recurrence, or the site of impaction. The region in which the stone was found in RGSI and IGSI was similar in most cases (*p*=0.002). Logistic regression also revealed a higher probability of stone impaction in the ileum in RGSI if it was the site of impaction in IGSI. In most cases, enterolithotomy was the preferred method.

**Conclusions:**

A high index of suspicion for RGSI should be maintained for older women with a history of GSI. The region where the stone was impacted during IGSI should be investigated first in such patients.

## 1. Introduction

Gallstone ileus (GSI) is a severe complication of cholelithiasis in which a stone enters the enteric lumen and causes mechanical obstruction [1–3]. Recurrent gallstone ileus (RGSI) occurs in 5–8% of patients with GSI [4]. The stone responsible for RGSI can emanate from an untreated biliary-enteric fistula with cholelithiasis, or it could be a gallstone that was not removed at the time of index operation [5]. RGSI increases the clinical dilemmas associated with the treatment of GSI, as the patient is now at increased risk and the prevention of further recurrences is a high priority [6].

The potential variables associated with RGSI are unknown mainly due to the lack of prospective or retrospective studies. However, fortunately, there are several case studies available that provide information on the demographic and clinical characteristics of RGSI and might be helpful in clinical decision-making [4–11]. In a significant attempt to clarify the clinical characteristics of patients with RGSI, Mir et al. [12] conducted a systematic review of the cases reported in the literature up to 2015. Although these authors provided a highly comprehensive and critical overview of the demographic and clinical characteristics of RGSI, their work did not report data synthesis and subgroup analysis.

The objective of this meta-analysis was to compile important clinical and demographic characteristics of patients with RGSI, both in index GSI (IGSI), that is, the first episode of GSI and in RGSI, and to examine the factors associated with mortality and SOI.

## 2. Methods

This review was conducted in accordance with the Preferred Reporting Items for Systematic Review and Meta-Analyses (PRISMA) guidelines (Table S1) [13]. As there were no prospective or retrospective studies or clinical trials on RGSI, the focus was on case reports and case series [14, 15]. The inclusion criteria were primarily based on the fact that the case must have a confirmed diagnosis of RGSI. Data elements such as (i) SOI in IGSI and RGSI, (ii) size of stone in IGSI and in RGSI, (iii) number of stones in IGSI and in RGSI, (iv) symptoms of RGSI, (v) time duration between IGSI and RGSI, (vi) predisposing factors, (vii) diagnosis modality (s), (viii) management strategies during IGSI and at RGSI, and (ix) complications/adverse events in RGSI (x) mortality were given high priority in the analysis. The SOI at IGSI was referred to as ISOI, whereas the SOI at RGSI was referred to as RSOI. Conference/symposium abstracts, letters to the editors, cases with incomplete descriptions, reviews, or case series with aggregate data with incomplete information on individual cases were excluded.

### 2.1. Search Strategies and Information Sources

A systematic search of key terms was carried out in the Scopus, Web of Science (WOS), PubMed, and Cochrane databases. The search was completed on July 14, 2020 (Table S2). The search terms used were the combination of “intestinal obstruction,” “gallstone,” or “GSI,” and “recurrent” or “recurrence.” Reference lists of the selected articles were also examined to find other relevant articles.

### 2.2. Study Selection and Definitions

The authors independently conducted the database search (NR and BI), with the initial results imported and processed using reference management software. All duplicates were removed first, and then the titles and abstracts of the articles were examined. The authors retrieved the full texts of the relevant articles and independently evaluated them (NR, BI, SA, and AA). Data were extracted using a standardized form containing 25 variables related to IGSI and RGSI. Data elements extracted included country, publication year, demographic data, comorbidities, diagnosis, SOI, and hospital mortality. Depending on the anatomical location, the SOI was divided into three broader categories: jejunum, ileum (ileum or ileocecal valve), and others. RGSI was defined as the intraoperatively/radiologically confirmed recurrence of intestinal obstruction by a gallstone. A faceted stone is one that has at least one flattened side. Multiple stones are defined by the presence of more than one stone; in the analyses, the number of stones was stratified as a single stone, two stones, or more than two stones. The duration of the symptoms was defined as the duration from the onset of the symptom to hospital admission. Recurrence time was defined as the duration in days between discharge after IGSI and readmission for RGSI; when RGSI occurred before discharge, the time difference between index episode and RGSI was considered recurrence time. Large stones were defined as having at least one dimension greater than 3 cm [16, 17]. Mortality was defined as the percentage of patients who died in a particular group.

### 2.3. Summary Measures and Statistical Analysis

The characteristics of the patients and surgical details were descriptively summarized. Statistical significance of categorical variables was examined by the chi-square test or Fischer's exact test and of continuous variables by the Wilcoxon, Mann–Whitney, or Kruskal–Wallis test. The risk factors for mortality and SOI in RGSI were analyzed using exploratory univariate and stepwise multivariate logistic regression. Subgroup analysis was also performed with respect to age, SOI, and sex. A *p* value of < 0.05 was considered statistically significant. Missing data were excluded from the respective analysis, and the total number of cases available for each analysis was provided as the denominator; for example, in 16/47, 47 represents the total number of patients for whom the respective information was available.

## 3. Results

### 3.1. Study Selection, Completeness of Data, and Demographics

**Figure 1:**
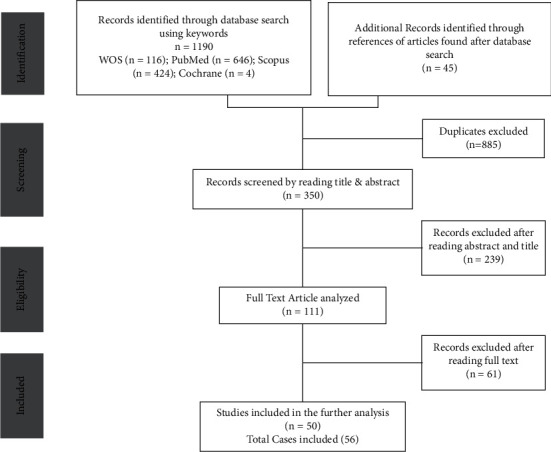
Flow diagram used for data extraction.

**Figure 2:**
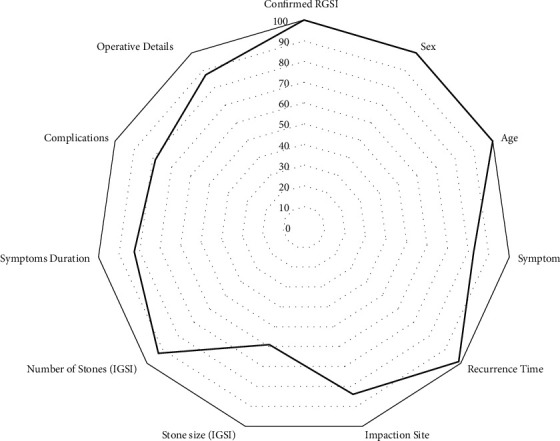
Completeness of data extraction.

In total, 1190 articles were obtained after the database search, and 45 additional records were identified after the screening of references; of these, 50 (56 individual cases) were included in the final analysis (Figure 1) [1, 5–8, 11, 18–61]. The data extraction efficiency for key variables was 89.9% (Figure 2).

### 3.2. IGSI: Clinical Characteristics and Management

In IGSI, the ileum was reported to be the most common SOI (Table 1), representing 61.7% (29/47) of all cases, followed by the jejunum (16/47, 34.0%) and others (2/47, 4.3%). For 33 patients, information on the size of the stone was available. In IGSI, a large primary stone (size ≥3 cm) was observed in 75.8% of patients (25/33). Only 18 cases explicitly specified the presence or absence of a faceted stone, and 66.7% of such cases (12/18) mentioned the occurrence of a faceted stone; however, the faceted stone in IGSI was not exclusively assigned as the cause of RGSI in most cases. In IGSI, the status of multiple stones was reported in 52 cases, with only 3 (3/52, 5.8%) of them having more than two stones.

Operative details of IGSI were available for 51 patients. Enterolithotomy alone was performed in 82.4% (42/51) of IGSI cases, enterolithotomy + segmental resection was used in 5 (5/51, 9.8%) patients [7, 18, 21, 24], and other interventions were used in only 4 (4/51, 7.8%) cases [20, 54, 61].

### 3.3. Time of Recurrence

**Figure 3:**
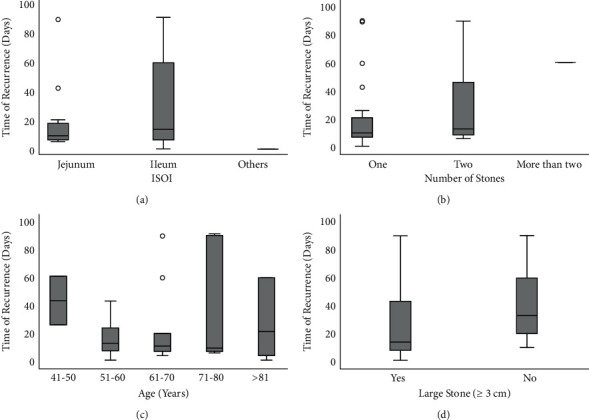
Time of recurrence, (a) ISOI (*p*=0.73), (b) number of stones (*p*=0.27), (c) age groups in years (*p*=0.96), and (d) size of stones (*p*=0.18).

The time of recurrence was evaluated as a function of the number of stones, the size of the stones, the age, and ISOI to determine whether any clinical manifestations of IGSI affect the time of recurrence (Figure 3). The median recurrence time (interquartile range, IQR) was 20.5 (8.5–95.5) days (Table 1; Figure S1). ISOI or the number of stones at IGSI did not have a statistically significant effect on the recurrence time (Table 2; Figures 3(a) and 3(b)). There were no statistically significant differences in the time of recurrence between patients of different ages (*p*=0.96, Figure 3(c)). The time of recurrence in patients with large stones was not statistically different from that in patients with smaller stones (*p*=0.18, Figure 3(d)). To eliminate the outlier effect, this study did not include the time of recurrence of more than 100 days. The association between age and time of recurrence was also investigated, but no correlation was found (*r* = 0.02, *p*=0.97, Figure S2).

### 3.4. RGSI: Clinical Characteristics and Management

The ileum was the most common SOI in RGSI, accounting for 62.0% of patients (31/50), and the jejunum was the SOI in 15 (15/50, 30.0%) patients (Table 1). Other SOIs were the duodenum [31] and the rectum [56, 57], represented in 1 and 3 cases, respectively. The median age was 70 years (interquartile range (IQR): 63–76 years), and 48/55 (87.3%) were women (Table 1). Most of the patients had symptoms such as abdominal pain, vomiting, or nausea. The median duration of symptoms was 2.5 (1.0–7.0) days. Patients reported abdominal discomfort, vomiting, and nausea in 93.6% (44/47), 80.9% (38/47), and 34.0% (16/47) of the cases, respectively. Operative details for the treatment of RGSI were available for 53 patients. In 79.2% of the patients (42/53), enterolithotomy was the surgical method. In 5 patients (5/53, 9.4%), enterolithotomy + cholecystectomy was performed [7, 18, 29, 32, 36] and enterolithotomy + segmental resection was used only in 2 (2/53, 3.8%) cases [33, 44]. Nonsurgical management was used in 4 (4/53, 7.5%) cases [20, 31, 56, 57]. The overall mortality rate was 11.8% (6/51). Five of the six deaths occurred after an enterolithotomy; however, in terms of mortality, there were no statistically significant differences between patients with RGSI who underwent different surgical procedures (*p*=0.32, Table S3). In 28.3% (13/46) of the cases, postoperative complications were observed. There was no statistically significant difference in the frequency of complications among patients with RGSI who underwent different surgical procedures (*p*=0.17, Table S3). Predictors of mortality in RGSI were investigated using univariate logistic regression; however, no statistically significant variables were identified (Table S4).

RGSI was not observed in patients under 40 years of age (Table S5). In fact, only 2 (3.6%) patients were between the ages of 41 and 50 years. The 51–60-year-old age group represented 18.2% of all patients. More than 75% of the patients were older than 60 years. Furthermore, no significant differences in clinical characteristics were found between men and women (Table S6).

### 3.5. Regularity of the SOI

**Figure 4:**
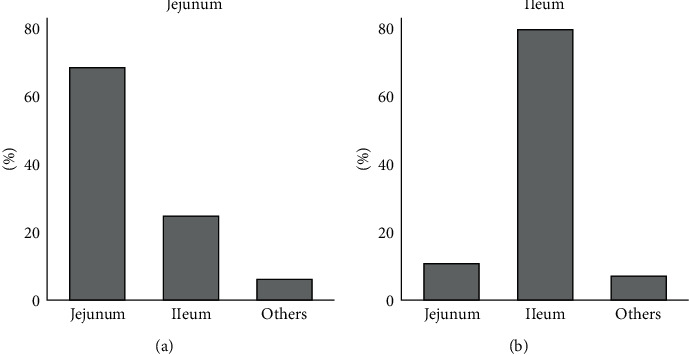
Impaction sites in RGSI patients, (a) with impaction in the jejunum during IGSI and (b) with impaction in the ileum during IGSI.

For 45 patients, matched data for SOI were available in IGSI and RGSI. In 11 of 16 cases (68.8%), in which ISOI was the jejunum, RSOI was also the jejunum. Furthermore, in 22 of 27 cases (81.5%) in which ISOI was the ileum, RSOI was also in the ileum (*p*=0.002, Figure 4 and Table 2).

A logistic regression analysis was performed to investigate the likely effect of various parameters observed during IGSI on RSOI (ileum); the variables used in the analysis were large stones at IGSI, single versus more than one stone during IGSI, age, ISOI (ileum), and sex. None of the variables except ISOI (ileum) had a statistically significant association with the RSOI (ileum) in univariate analysis. Multiple stepwise logistic regression was performed for large stones, sex, and ISOI (ileum). The overall model correctly predicted 84.6% of the cases. Only ISOI (ileum) was found to have a significant association with RSOI (ileum) (odds ratio: 36.5, 95% confidence interval (CI): 2.5–532.6, *p*=0.009, Table 3). The *p* value for the Hosmer–Lemeshow goodness-of-fit statistic was 0.56, and the area under the ROC curve was 0.884.

## 4. Discussion

This study presented the first meta-analysis of RGSI. This analysis provided information on the key aspects of RGSI, such as SOI in IGSI and RGSI, the pattern of surgical interventions, age- and sex-stratified analysis, the correlation between age and recurrence time, mortality, and morbidity. A systematic review of RGSI is available in cases reported up to April 2015 [12]; however, a pooled analysis of important characteristics of RGSI is missing from the literature. This study complements, extends, and updates the previous systematic review by conducting a meta-analysis of case reports.

Currently, there are no standard guidelines for the management of GSI. When GSI recurs, the dilemmas associated with its management become considerably more complicated because the patient has already undergone surgery [1, 4, 6, 7, 12, 22, 23, 25, 62–65]. In most cases, the recurrence happens within a few days of the IGSI; thus, there is not much time between index admission and readmission for the patient's physical and psychological recuperation. The pooled mortality rate in RGSI was determined to be 11.8% in this study. Age is frequently seen as a risk factor for mortality and surgical complications [18]; however, this study did not observe an increased propensity to mortality or morbidity in patients in relatively higher age groups. These findings do not necessarily rule out the possibility of a risk associated with advanced age or a surgical procedure; however, they may indicate that the respective surgeons have already implemented appropriate care and surgical selection, which take into account the risks associated with age and surgery, resulting in minimal age-group-dependent variations in mortality and morbidity [66]. Notably, due to the high rate of postoperative morbidity and mortality in a single-step approach (enterolithotomy + cholecystectomy)), most surgeons prefer the enterolithotomy only approach in GSI [6, 9, 12, 22, 23, 25, 64, 66–69].

This study also indicated that most of the patients with RGSI were women, which confirms that women are at a higher risk for RGSI. The ileum was the SOI most frequently observed in both IGSI and RGSI. This observation is consistent with other findings [5, 23, 31, 34, 35, 63, 67, 70–73]. However, one notable aspect was that SOI was identical in approximately 70% of cases in IGSI and RGSI; that is, in RGSI, the ileum was not the predominant SOI in patients who had impaction at the jejunum in IGSI. Multivariate logistic regression also confirmed these findings. It should also be noted that there were no statistically significant differences between SOI and the observed incidence of large stones. More research is needed to substantiate our findings; however, when there is a suspicion of RGSI, it may be beneficial to focus on the region where the stone was impacted in the IGSI. Given the dilemma of diagnosing and managing emergencies in RGSI, such a method may provide a practical advantage.

Conservative management was adopted in only four cases. RSOI was the rectum in two of these cases [56, 57], and in one case, SOI was the duodenum and the management approach involved Holmium: YAG laser lithotripsy [31]. RGSI is often treated with enterolithotomy or with cholecystectomy and repair of the enteric biliary fistula [72, 74, 75]. Our analysis found that enterolithotomy was the procedure most commonly used in approximately 82% and 80% of cases at IGSI and RGSI, respectively. Furthermore, SOI had little effect on the surgical procedures chosen, demonstrating a common predilection for enterolithotomy [1, 6, 7, 24, 27, 69, 76].

The current systematic review provides the first meta-analysis and the most up-to-date state of the epidemiological and clinical aspects of RGSI. The cases included in this paper were identified by a thorough search of the major databases using the PRISMA approach, and only case reports with good reporting standards were included. However, despite carefully applying a rigorous inclusion criterion, it is impossible to rule out the possibility of missing some cases aggregated in case series, those that were not published in English, or those reported in conference abstract or letter to the editor. Furthermore, patients with missing data were excluded from the analysis, further limiting the generalizability of the synthesized data. Publication bias, which is generally observed in the publication of a case report, can also affect the generalizability of our results. Furthermore, the cases reported in the analysis cover a broad period; therefore, temporal variations with advances in diagnostic modalities and treatment and their corresponding impact on clinical outcomes are quite probable. Such factors should be considered before generalizing the results. It may be noted that, generally, the rarest and clinically challenging cases are reported and accepted as case reports; therefore, common or less significant cases may be underrepresented. A lower number of cases also caution the generalization of the results reported in this work. Despite these limitations, the results of this meta-analysis reveal more nuanced details of patients with RGSI, which may help in the management of this rare and high-risk condition.

## 5. Conclusions

The findings of this meta-analysis have helped to determine various unexplored aspects of RGSI. Particularly notable findings are that more than 60-year-old women represent most cases of RGSI, and SOI in IGSI and RGSI are frequently similar. Recurrence time is not correlated with age or with ISOI, stone size at IGSI, or sex. Future research should focus on the pathophysiology of stone formation, migration dynamics, and identifiable anatomical features of the small intestine.

## Figures and Tables

**Table 1:** Demographic and clinical characteristics of patients.

	Variable	#Summary *n* (%) or median (Q1, Q3)
IGSI	ISOI (*N* = 47)	
	Jejunum	16 (34.0%)
	Ileum	29 (61.7%)
	Others	2 (4.3%)
	Stone ≥ 3 cm (*N* = 33)	25 (75.8%)
	Faceted stone (*N* = 18)	12 (66.7%)
	Number of stones (*N* = 52)	
	One	41 (78.8%)
	Two	8 (15.4%)
	More than two	3 (5.8%)
	Surgery (*N* = 51)	
	Enterolithotomy	42 (82.4%)
	Enterolithotomy + segmental resection	5 (9.8%)
	Others	4 (7.8%)

RGSI	Time of recurrence (days) (*N* = 56)	20.5 (8.5, 95.5)
	RSOI (*N* = 50)	
	Jejunum	15 (30.0%)
	Ileum	31 (62.0%)
	Others	4 (8.0%)
	Age (years) (*N* = 55)	70.0 (63.0, 76.0)
	Sex (women) (*N* = 55)	48 (87.3%)
	Symptoms (RGSI) (*N* = 47)	
	Abdominal pain	44 (93.6%)
	Vomit	38 (80.9%)
	Nausea	16 (34.0%)
	Constipation	5 (10.6%)
	Symptom duration (RGSI) (*N* = 18)	2.5 (1.0, 7.0)
	Surgery (RGSI) (*N* = 53)	
	Enterolithotomy	42 (79.2%)
	Enterolithotomy + cholecystectomy	5 (9.4%)
	Enterolithotomy + segmental resection	2 (3.8%)
	Others	4 (7.5%)
	Mortality (RGSI) (*N* = 51)	6 (11.8%)
	Complications (RGSI) (*N* = 46)	13 (28.3%)

^#^Continuous variables are presented as median and IQR and categorical variables as number (*n*) and (percentage); *N* represents the total number of patients for whom the data was available for that particular variable; IQR: interquartile range; GSI: gallstone ileus; RGSI: recurrent gallstone ileus; IGSI: index gallstone ileus; ISOI: site of impaction in index gallstone ileus; RSOI: site of impaction in recurrent gallstone ileus.

**Table 2:** ISOI and clinical characteristics of patients in IGSI and RGSI.

	Variable	ISOI				*p* value
		Jejunum	Ileum	Others	Total	
IGSI	Stone ≥ 3 cm (*N* = 32)					0.730
	Yes	9 (75.0%)	14 (77.8%)	2 (100.0%)	25 (78.1%)	
	No	3 (25.0%)	4 (22.2%)	0 (0.0%)	7 (21.9%)	
	Faceted stone (*N* = 17)					0.602
	Yes	5 (71.4%)	5 (55.6%)	1 (100.0%)	11 (64.7%)	
	No	2 (28.6%)	4 (44.4%)	0 (0.0%)	6 (35.3%)	
	Number of stones (*N* = 46)					0.670
	One	14 (87.5%)	22 (78.6%)	2 (100.0%)	38 (82.6%)	
	Two	2 (12.5%)	3 (10.7%)	0 (0.0%)	5 (10.9%)	
	More than two	0 (0.0%)	3 (10.7%)	0 (0.0%)	3 (6.5%)	
	Surgery (*N* = 44)					
	Enterolithotomy	14 (87.5%)	23 (88.5%)	1 (50.0%)	38 (86.4%)	
	Enterolithotomy + segmental resection	2 (12.5%)	3 (11.5%)	0 (0.0%)	5 (11.4%)	
	Others	0 (0.0%)	0 (0.0%)	1 (50.0%)	1 (2.3%)	

RGSI	Sex (*N* = 47)					0.852
	Men	2 (12.5%)	4 (13.8%)	0 (0.0%)	6 (12.8%)	
	Women	14 (87.5%)	25 (86.2%)	2 (100.0%)	41 (87.2%)	
	Age (years) (*N* = 47)	71.5 (65.0, 76.0)	69.0 (67.0, 76.0)	59.0 (55.0, 63.0)	70.0 (65.0, 76.0)	0.200
	Time of recurrence (*N* = 47)	15.0 (7.5, 97.5)	26.0 (9.0, 91.0)	50.4 (0.8, 100.0)	20.0 (8.0, 100.0)	0.738
	Symptoms (*N* = 41)					
	Abdominal pain					0.903
	Yes	14 (93.3%)	22 (91.7%)	2 (100.0%)	38 (92.7%)	
	No	1 (6.7%)	2 (8.3%)	0 (0.0%)	3 (7.3%)	
	Vomit					0.520
	Yes	13 (86.7%)	18 (75.0%)	2 (100.0%)	33 (80.5%)	
	No	2 (13.3%)	6 (25.0%)	0 (0.0%)	8 (19.5%)	
	RSOI (*N* = 45)					0.002
	Jejunum	11 (68.8%)	3 (11.1%)	0 (0.0%)	14 (31.1%)	
	Ileum	4 (25.0%)	22 (81.5%)	2 (100.0%)	28 (62.2%)	
	Others	1 (6.3%)	2 (7.4%)	0 (0.0%)	3 (6.7%)	
	Stone ≥ 3 cm (*N* = 31)					0.028
	Yes	7 (53.8%)	15 (93.8%)	2 (100.0%)	24 (77.4%)	
	No	6 (46.2%)	1 (6.3%)	0 (0.0%)	7 (22.6%)	
	Surgery (*N* = 45)					0.994
	Enterolithotomy	13 (81.3%)	23 (85.2%)	2 (100.0%)	38 (84.4%)	
	Enterolithotomy + cholecystectomy	1 (6.3%)	2 (7.4%)	0 (0.0%)	3 (6.7%)	
	Enterolithotomy + segmental resection	1 (6.3%)	1 (3.7%)	0 (0.0%)	2 (4.4%)	
	Others	1 (6.3%)	1 (3.7%)	0 (0.0%)	2 (4.4%)	

^#^Continuous variables are presented as median and IQR and categorical variables as number (*n*) and percentage; *N* represents the total number of patients for whom data were available for that particular variable; IGSI: index gallstone ileus; RGSI: recurrent gallstone ileus; ISOI: site of impaction in index gallstone ileus; RSOI: site of impaction in RGSI; categorical variables were examined by the chi-square test and continuous variables by the Kruskal–Wallis test.

**Table 3:** Logistic regression analysis of the factors affecting the risk of RSOI (ileum).

Variable	OR	Univariate analysis		*p* value	OR	Multivariate analysis		*p* value
		95% CI				95% CI		
		Lower	Upper			Lower	Upper	
Large stone (IGSI)	0.19	0.03	1.30	0.091	0.17	0.01	3.01	0.225
Sex (women)	1.44	0.21	9.66	0.37	8.35	0.21	324.6	0.256
Number of stones (IGSI)	1.03	0.30	3.57	0.97				
Age	0.98	0.93	1.04	0.543				
ISOI (ileum)	20.2	3.8	106.4	<0.001	36.5	2.50	532.6	0.009

The area under the ROC curve: 0.884; Hosmer–Lemeshow goodness-of-fit statistic *p* value = 0.56; multicollinearity was not found among the independent variables used for multivariate analysis. RSOI: site of impaction at the RGSI; RGSI: recurrent gallstone ileus; OR: odds ratio; CI: confidence interval; ISOI: site of impaction at the IGSI; IGSI: index gallstone ileus.

## Data Availability

The data used to support the findings of this study are available from the corresponding author upon request.

## Supplementary Materials

Supplementary Materials Supplementary tables include details of PRISMA checklist, surgery type, mortality, adverse events at RGSI, and demographics and clinical features of patients in different age groups. Supplementary figures include figures related to spread of time of recurrence (days), spread of symptoms duration (days), and correlation between age and time of recurrence (days).
