# Supplementary material for: The Regularity of the Site of Impaction in Recurrent Gallstone Ileus: A Systematic Review and Meta-Analysis of Reported Cases
Source: Can J Gastroenterol Hepatol. 2021 Dec 2;2021:5539789. doi: 10.1155/2021/5539789 (PMC8660221; doi:10.1155/2021/5539789)
Supplement: Supplementary Materials — Supplementary tables include details of PRISMA checklist, surgery type, mortality, adverse events at RGSI, and demographics and clinical features of patients in different age groups. Supplementary figures include figures related to spread of time of recurrence (days), spread of symptoms duration (days), and correlation between age and time of recurrence (days). [file 5539789.f1.zip › 5539789.f1/Suppl_figureR3.docx]

**Supplementary Figures**


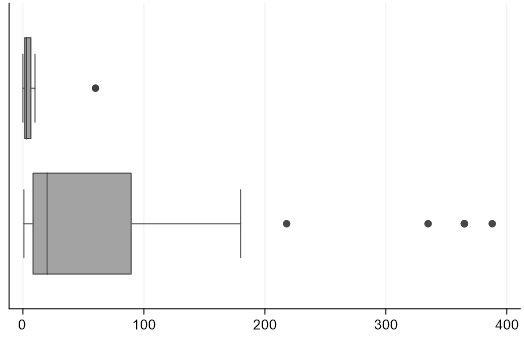


(B)

(A)

Figure S1: (A) Spread of time of recurrence (Days) (B) Spread of symptoms duration (Days)

Figure S2: Correlation between age and time of recurrence (days)
