# Supplementary material for: The Regularity of the Site of Impaction in Recurrent Gallstone Ileus: A Systematic Review and Meta-Analysis of Reported Cases
Source: Can J Gastroenterol Hepatol. 2021 Dec 2;2021:5539789. doi: 10.1155/2021/5539789 (PMC8660221; doi:10.1155/2021/5539789)
Supplement: Supplementary Materials — Supplementary tables include details of PRISMA checklist, surgery type, mortality, adverse events at RGSI, and demographics and clinical features of patients in different age groups. Supplementary figures include figures related to spread of time of recurrence (days), spread of symptoms duration (days), and correlation between age and time of recurrence (days). [file 5539789.f1.zip › 5539789.f1/Suppl_tablesR3.docx]

Supplementary Tables

Table S1: PRISMA checklist

| Section/topic | # | Checklist item | Reported on page # |
| --- | --- | --- | --- |
| TITLE | | |  |
| Title | 1 | Identify the report as a systematic review, meta-analysis, or both. | 1 |
| ABSTRACT | | |  |
| Structured summary | 2 | Provide a structured summary including, as applicable: background; objectives; data sources; study eligibility criteria, participants, and interventions; study appraisal and synthesis methods; results; limitations; conclusions and implications of key findings; systematic review registration number. | 2 |
| INTRODUCTION | | |  |
| Rationale | 3 | Describe the rationale for the review in the context of what is already known. | 3 |
| Objectives | 4 | Provide an explicit statement of questions being addressed with reference to participants, interventions, comparisons, outcomes, and study design (PICOS). | 3, as appropriate for a case reports based meta-analysis. |
| METHODS | | |  |
| Protocol and registration | 5 | Indicate if a review protocol exists, if and where it can be accessed (e.g., Web address), and, if available, provide registration information including registration number. | Not Available |
| Eligibility criteria | 6 | Specify study characteristics (e.g., PICOS, length of follow-up) and report characteristics (e.g., years considered, language, publication status) used as criteria for eligibility, giving rationale. | 4 |
| Information sources | 7 | Describe all information sources (e.g., databases with dates of coverage, contact with study authors to identify additional studies) in the search and date last searched. | 4-5 |
| Search | 8 | Present full electronic search strategy for at least one database, including any limits used, such that it could be repeated. | 38 |
| Study selection | 9 | State the process for selecting studies (i.e., screening, eligibility, included in systematic review, and, if applicable, included in the meta-analysis). | 4-5 |
| Data collection process | 10 | Describe method of data extraction from reports (e.g., piloted forms, independently, in duplicate) and any processes for obtaining and confirming data from investigators. | 4-5 |
| Data items | 11 | List and define all variables for which data were sought (e.g., PICOS, funding sources) and any assumptions and simplifications made. | 4-5 |
| Risk of bias in individual studies | 12 | Describe methods used for assessing risk of bias of individual studies (including specification of whether this was done at the study or outcome level), and how this information is to be used in any data synthesis. | Data completeness was assessed, Page 24. as appropriate for case reports based meta-analysis. |
| Summary measures | 13 | State the principal summary measures (e.g., risk ratio, difference in means). | 5 |
| Synthesis of results | 14 | Describe the methods of handling data and combining results of studies, if done, including measures of consistency (e.g., I^2^) for each meta-analysis. | 5, as applicable for case reports. |

Table S2: Search strategy and hits

| Keyword | PubMed | WOS | SCOPUS | Cochrane |
| --- | --- | --- | --- | --- |
|  | Hit | Hit | Hit | Hit |
| Recurrent gallstone intestinal obstruction | 137 | 14 | 84 | 2 |
| recurrence gallstone intestinal obstruction | 137 | 7 | 61 | 2 |
| Recurrent gallstone ileus | 128 | 47 | 125 | 0 |
| Recurrence gallstone ileus | 128 | 14 | 87 | 0 |
| GSI recurrence | 58 | 19 | 41 | 0 |
| GSI recurrent | 58 | 15 | 26 | 0 |
| Grand Total | 1190 | | | |

WOS: Web of Science

Table S3: Surgery type, mortality, and adverse events at RGSI

| Surgery | Mortality | | Complications | |
| --- | --- | --- | --- | --- |
|  | Total cases* | Deaths, n (%) | Total cases* | Complications, n (%) |
| Enterolithotomy | 41 | 5 (12.2) | 38 | 10(26.3) |
| Enterolithotomy+ cholecystectomy | 5 | 0(0) | 5 | 1(20.0) |
| Enterolithotomy+ segmental resection | 2 | 1(50) | 1 | 1(100.0) |
| Others | 3 | 0(0) | 2 | 1(50.0) |
| p-value | 0.32 | | 0.17 | |

* Total cases represent the number of patients for whom the respective data was available, mortality and complication data were available for 51 and 46 patients, respectively; RGSI: recurrent gallstone ileus; the chi-square test was used compare the variables, and the p-value was calculated for the comparison of four surgery groups.

Table S4: Univariate logistic regression analysis for mortality after surgery for RGSI

|  | Odds Ratio | 95% C.I. for Odd ratio | | p-value |
| --- | --- | --- | --- | --- |
|  |  | Lower | Upper |  |
| Age | 1.02 | 0.95 | 1.10 | 0.59 |
| Time of recurrence (Days) | 1.00 | 0.98 | 1.01 | 0.46 |
| RSOI | 0.37 | 0.07 | 1.78 | 0.21 |
| Multiple stones (RGSI) | 2.71 | 0.41 | 17.7 | 0.29 |
|  |  |  |  |  |

C.I. Confidence Interval; RSOI: site of impaction at the RGSI; RGSI: recurrent gallstone ileus. All observed cases of mortality were in women and involved large stones. These variables therefore, could not be used in logistic regression.

Table S5: Demographic and clinical features of patients in different age-groups

| Variable | Age group | | | | | | p-value |
| --- | --- | --- | --- | --- | --- | --- | --- |
|  | 41-50 | 51-60 | 61-70 | 71-80 | >81 | Total |  |
| Sex |  |  |  |  |  |  | 0.943 |
| Men | 0 (0.0%) | 1 (10.0%) | 2 (11.8%) | 3 (17.6%) | 1 (11.1%) | 7 (12.7%) |  |
| Women | 2 (100.0%) | 9 (90.0%) | 15 (88.2%) | 14 (82.4%) | 8 (88.9%) | 48 (87.3%) |  |
| ISOI |  |  |  |  |  |  | 0.646 |
| Jejunum | 0 (0.0%) | 2 (33.3%) | 5 (31.3%) | 7 (46.7%) | 2 (25.0%) | 16 (34.0%) |  |
| Ileum | 2 (100.0%) | 3 (50.0%) | 10 (62.5%) | 8 (53.3%) | 6 (75.0%) | 29 (61.7%) |  |
| Other | 0 (0.0%) | 1 (16.7%) | 1 (6.3%) | 0 (0.0%) | 0 (0.0%) | 2 (4.3%) |  |
| Stone ≥3cm (IGSI) |  |  |  |  |  |  | 0.066 |
| Yes | 2 (100.0%) | 6 (100.0%) | 9 (75.0%) | 6 (85.7%) | 2 (33.3%) | 25 (75.8%) |  |
| No | 0 (0.0%) | 0 (0.0%) | 3 (25.0%) | 1 (14.3%) | 4 (66.7%) | 8 (24.2%) |  |
| Faceted Stone (IGSI) |  |  |  |  |  |  | 0.507 |
| Yes | 0 (0.0%) | 4 (80.0%) | 4 (57.1%) | 3 (75.0%) | 1 (100.0%) | 12 (66.7%) |  |
| No | 1 (100.0%) | 1 (20.0%) | 3 (42.9%) | 1 (25.0%) | 0 (0.0%) | 6 (33.3%) |  |
| Number of Stones (IGSI) |  |  |  |  |  |  | 0.038 |
| One | 1 (50.0%) | 4 (57.1%) | 16 (94.1%) | 13 (81.3%) | 6 (66.7%) | 40 (78.4%) |  |
| Two | 0 (0.0%) | 3 (42.9%) | 0 (0.0%) | 3 (18.8%) | 2 (22.2%) | 8 (15.7%) |  |
| More than two | 1 (50.0%) | 0 (0.0%) | 1 (5.9%) | 0 (0.0%) | 1 (11.1%) | 3 (5.9%) |  |
| Surgery (IGSI) |  |  |  |  |  |  | 0.632 |
| Enterolithotomy | 1 (50.0%) | 8 (88.9%) | 13 (81.3%) | 13 (81.3%) | 7 (87.5%) | 42 (82.4%) |  |
| Enterolithotomy +  Segmental resection | 1 (50.0%) | 0 (0.0%) | 2 (12.5%) | 2 (12.5%) | 0 (0.0%) | 5 (9.8%) |  |
| Others | 0 (0.0%) | 1 (11.1%) | 1 (6.3%) | 1 (6.3%) | 1 (12.5%) | 4 (7.8%) |  |
| Time of Recurrence |  |  |  |  |  |  | 0.960 |
| Median (Q1, Q3) | 43.5 (26.0, 61.0) | 17.0 (10.0, 43.0) | 20.0 (10.0, 124.0) | 11.0 (7.0, 90.0) | 60.0 (10.0, 104.0) | 21.0 (9.0, 100.0) |  |
| Symptoms (RGSI) |  |  |  |  |  |  |  |
| Abdominal Pain |  |  |  |  |  |  | 0.010 |
| Yes | 1 (50.0%) | 6 (75.0%) | 14 (100.0%) | 13 (100.0%) | 9 (100.0%) | 43 (93.5%) |  |
| No | 1 (50.0%) | 2 (25.0%) | 0 (0.0%) | 0 (0.0%) | 0 (0.0%) | 3 (6.5%) |  |
| Vomit |  |  |  |  |  |  | 0.009 |
| Yes | 0 (0.0%) | 5 (62.5%) | 14 (100.0%) | 11 (84.6%) | 7 (77.8%) | 37 (80.4%) |  |
| No | 2 (100.0%) | 3 (37.5%) | 0 (0.0%) | 2 (15.4%) | 2 (22.2%) | 9 (19.6%) |  |
| Nausea |  |  |  |  |  |  | 0.908 |
| Yes | 1 (50.0%) | 2 (25.0%) | 5 (35.7%) | 4 (30.8%) | 4 (44.4%) | 16 (34.8%) |  |
| No | 1 (50.0%) | 6 (75.0%) | 9 (64.3%) | 9 (69.2%) | 5 (55.6%) | 30 (65.2%) |  |
| Constipation |  |  |  |  |  |  | 0.694 |
| Yes | 0 (0.0%) | 2 (25.0%) | 1 (7.1%) | 1 (7.7%) | 1 (11.1%) | 5 (10.9%) |  |
| No | 2 (100.0%) | 6 (75.0%) | 13 (92.9%) | 12 (92.3%) | 8 (88.9%) | 41 (89.1%) |  |
| RSOI |  |  |  |  |  |  | 0.574 |
| Jejunum | 0 (0.0%) | 1 (12.5%) | 5 (29.4%) | 8 (50.0%) | 1 (14.3%) | 15 (30.0%) |  |
| Ileum | 2 (100.0%) | 6 (75.0%) | 11 (64.7%) | 7 (43.8%) | 5 (71.4%) | 31 (62.0%) |  |
| Others | 0 (0.0%) | 1 (12.5%) | 1 (5.9%) | 1 (6.3%) | 1 (14.3%) | 4 (8.0%) |  |
| Stone ≥ 3cm (RGSI) |  |  |  |  |  |  | 0.608 |
| Yes | 2 (100.0%) | 4 (66.7%) | 11 (84.6%) | 4 (57.1%) | 5 (71.4%) | 26 (74.3%) |  |
| No | 0 (0.0%) | 2 (33.3%) | 2 (15.4%) | 3 (42.9%) | 2 (28.6%) | 9 (25.7%) |  |
| Number of Stones (RGSI) |  |  |  |  |  |  | 0.262 |
| One | 1 (50.0%) | 9 (100.0%) | 13 (76.5%) | 14 (87.5%) | 6 (66.7%) | 43 (81.1%) |  |
| Two | 1 (50.0%) | 0 (0.0%) | 4 (23.5%) | 2 (12.5%) | 3 (33.3%) | 10 (18.9%) |  |
| Surgery (RGSI) |  |  |  |  |  |  | 0.507 |
| Enterolithotomy | 1 (50.0%) | 7 (77.8%) | 14 (82.4%) | 13 (81.3%) | 7 (77.8%) | 42 (79.2%) |  |
| Enterolithotomy+ Cholecystectomy | 1 (50.0%) | 2 (22.2%) | 1 (5.9%) | 1 (6.3%) | 0 (0.0%) | 5 (9.4%) |  |
| Enterolithotomy + Segmental resection | 0 (0.0%) | 0 (0.0%) | 1 (5.9%) | 1 (6.3%) | 0 (0.0%) | 2 (3.8%) |  |
| Others | 0 (0.0%) | 0 (0.0%) | 1 (5.9%) | 1 (6.3%) | 2 (22.2%) | 4 (7.5%) |  |
| Mortality (RGSI) |  |  |  |  |  |  | 0.986 |
| Yes | 0 (0.0%) | 1 (10.0%) | 2 (11.8%) | 2 (12.5%) | 1 (14.3%) | 6 (11.5%) |  |
| No | 2 (100.0%) | 9 (90.0%) | 15 (88.2%) | 14 (87.5%) | 6 (85.7%) | 46 (88.5%) |  |
| Complications (RGSI) |  |  |  |  |  |  | 0.406 |
| Yes | 1 (50.0%) | 1 (11.1%) | 3 (21.4%) | 4 (28.6%) | 4 (50.0%) | 13 (27.7%) |  |
| No | 1 (50.0%) | 8 (88.9%) | 11 (78.6%) | 10 (71.4%) | 4 (50.0%) | 34 (72.3%) |  |

#Continuous variables are presented as median and IQR, and categorical variables as number (n) and percentage (%); IGSI: index gallstone ileus; RGSI: recurrent gallstone ileus; categorical variables were examined by chi-square test and continuous variables by the Kruskal-Wallis test.

Table S6: Demographic and clinical features of men and women with RGSI

|  | Men | Women | Total | p-value |
| --- | --- | --- | --- | --- |
| Age (Years) |  |  |  | 0.480 |
| Median (Q1, Q3) | 73.0 (67.0, 77.0) | 69.5 (63.0, 76.0) | 70.0 (63.0, 76.0) |  |
| Time of Recurrence |  |  |  | 0.391 |
| Median (Q1, Q3) | 20.0 (9.0, 33.0) | 28.0 (8.5, 104.5) | 21.0 (9.0, 100.0) |  |
| ISOI |  |  |  | 0.852 |
| Jejunum | 2 (33.3%) | 14 (34.1%) | 16 (34.0%) |  |
| Ileum | 4 (66.7%) | 25 (61.0%) | 29 (61.7%) |  |
| Other | 0 (0.0%) | 2 (4.9%) | 2 (4.3%) |  |
| Faceted Stone (IGSI) |  |  |  | 0.289 |
| Yes | 2 (100.0%) | 10 (62.5%) | 12 (66.7%) |  |
| No | 0 (0.0%) | 6 (37.5%) | 6 (33.3%) |  |
| Number of Stones (IGSI) |  |  |  | 0.809 |
| One | 5 (83.3%) | 35 (77.8%) | 40 (78.4%) |  |
| Two | 1 (16.7%) | 7 (15.6%) | 8 (15.7%) |  |
| More than two | 0 (0.0%) | 3 (6.7%) | 3 (5.9%) |  |
| Surgery (IGSI) |  |  |  | 0.101 |
| Enterolithotomy | 4 (66.7%) | 38 (84.4%) | 42 (82.4%) |  |
| Enterolithotomy + Segmental resection | 2 (33.3%) | 3 (6.7%) | 5 (9.8%) |  |
| Others | 0 (0.0%) | 4 (8.9%) | 4 (7.8%) |  |
| Symptoms (RGSI) |  |  |  |  |
| Abdominal Pain |  |  |  | 0.532 |
| Yes | 5 (100.0%) | 38 (92.7%) | 43 (93.5%) |  |
| No | 0 (0.0%) | 3 (7.3%) | 3 (6.5%) |  |
| Vomit |  |  |  | 0.979 |
| Yes | 4 (80.0%) | 33 (80.5%) | 37 (80.4%) |  |
| No | 1 (20.0%) | 8 (19.5%) | 9 (19.6%) |  |
| Nausea |  |  |  | 0.462 |
| Yes | 1 (20.0%) | 15 (36.6%) | 16 (34.8%) |  |
| No | 4 (80.0%) | 26 (63.4%) | 30 (65.2%) |  |
| Constipation |  |  |  | 0.487 |
| Yes | 1 (20.0%) | 4 (9.8%) | 5 (10.9%) |  |
| No | 4 (80.0%) | 37 (90.2%) | 41 (89.1%) |  |
| RSOI |  |  |  | 0.662 |
| Jejunum | 2 (33.3%) | 13 (29.5%) | 15 (30.0%) |  |
| Ileum | 3 (50.0%) | 28 (63.6%) | 31 (62.0%) |  |
| Others | 1 (16.7%) | 3 (6.8%) | 4 (8.0%) |  |
| Stone ≥ 3cm (RGSI) |  |  |  | 0.972 |
| Yes | 3 (75.0%) | 23 (74.2%) | 26 (74.3%) |  |
| No | 1 (25.0%) | 8 (25.8%) | 9 (25.7%) |  |
| Surgery (RGSI) |  |  |  | 0.220 |
| Enterolithotomy | 4 (57.1%) | 38 (82.6%) | 42 (79.2%) |  |
| Enterolithotomy+ Cholecystectomy | 2 (28.6%) | 3 (6.5%) | 5 (9.4%) |  |
| Enterolithotomy + Segmental resection | 0 (0.0%) | 2 (4.3%) | 2 (3.8%) |  |
| Others | 1 (14.3%) | 3 (6.5%) | 4 (7.5%) |  |
| Mortality (RGSI) |  |  |  | 0.304 |
| No | 7 (100.0%) | 39 (86.7%) | 46 (88.5%) |  |
| Yes | 0 (0.0%) | 6 (13.3%) | 6 (11.5%) |  |
| Complications (RGSI) |  |  |  | 0.519 |
| Yes | 1 (16.7%) | 12 (29.3%) | 13 (27.7%) |  |
| No | 5 (83.3%) | 29 (70.7%) | 34 (72.3%) |  |
|  | | | | |

^#^Continuous variables are presented as median and IQR, and categorical variables as number (n) and percentage (%); IGSI: index gallstone ileus; RGSI: recurrent gallstone ileus; categorical variables were examined by chi-square test and continuous variables by the Mann-Whitney test.
